# Exploring the Impact of COVID-19 on Mental Health Outcomes in Children and Adolescents: A Systematic Review

**DOI:** 10.3390/ijerph17228479

**Published:** 2020-11-16

**Authors:** Finiki Nearchou, Clodagh Flinn, Rachel Niland, Sheena Siva Subramaniam, Eilis Hennessy

**Affiliations:** 1 School of Psychology, University College Dublin, 4 Dublin, Ireland; clodagh.flinn@ucdconnect.ie (C.F.); rachel.niland@ucdconnect.ie (R.N.); eilis.hennessy@ucd.ie (E.H.); 2 School of Medicine, University College Dublin, 4 Dublin, Ireland; sheena.sivasubramaniam@ucdconnect.ie

**Keywords:** children, adolescents, mental health, depression, anxiety, COVID-19, psychological impact, youth, pandemic

## Abstract

The psychological impact of the COVID-19 pandemic has been widely discussed during the past few months, with scholars expressing concern about its potential debilitating consequences on youth mental health. Hence, this research aimed to provide a systematic review of the evidence on the COVID-19 pandemic’s impact on youth mental health. We conducted a mixed methods integrated review to identify any empirical study that focused on young people ≤ 18 years old. Eight databases were systematically searched to identify studies of any type of research design. The selection procedure followed the Preferred Reporting Items for Systematic Review and Meta-Analyses (PRISMA) guidelines. The protocol of this systematic review was registered with PROSPERO (protocol ID: CRD4202019375). Twelve studies deemed eligible for data extraction (*n* = 12,262). The findings show that COVID-19 has an impact on youth mental health and is particularly associated with depression and anxiety in adolescent cohorts. The quality appraisal indicated that all studies were of low or moderate methodological quality. The COVID-19 pandemic is affecting young people’s lives, and thus generating robust research evidence to inform policy decisions is essential. Hence, the methodological quality of future research should be drastically improved.

## 1. Introduction

From December 2019 to 11 October 2020, the 2019 novel coronavirus (COVID-19) saw over 37 million confirmed cases and one million deaths globally, with almost half of these cases and deaths being reported in the region of the Americas and with the highest recent increase in the virus reported in the European region [1]. COVID-19 is associated with respiratory illness, occasionally leading to severe pneumonia and acute respiratory distress syndrome. Although comparisons have been made between this novel coronavirus and the 2003 severe acute respiratory syndrome (SARS), it has been suggested that due to the less severe clinical picture of COVID-19, the virus can spread more easily in the community than either SARS or Middle East Respiratory Syndrome (MERS) [2]. The lower fatality rate of COVID-19 means many more people are likely to survive their illness and have to face the psychological consequences.

### 1.1. Psychosocial Consequences

There is ample evidence from previous epidemics that individuals who recover from acute viral illnesses may experience significant mental distress and go on to experience psychiatric problems. For example, the immediate aftermath of the SARS epidemic saw the emergence of various psychiatric comorbidities, with the most common presenting problems involving increased levels of anxiety, depression, and features of post-traumatic stress reactions [3,4,5,6]. There is also evidence that these problems can be long lasting, with a study [7] reporting that 30 months following the SARS outbreak, psychiatric problems persisted for one third of those who had been infected. Nor is it only those who are infected by a virus that experience negative psychosocial consequences. Evidence from Canada suggested that healthcare workers experienced intense emotional reactions during the SARS outbreak, including fear of contagion, feelings of stigmatisation, boredom, loneliness, anger, anxiety and uncertainty [8,9].

Despite evidence of the negative impact of viral infection and quarantine on adults, there has been very limited research on their impact on children or adolescents. The evidence that exists focuses primarily on children with direct experience of illness or of quarantine in hospital as a result of contact with infected individuals. For example, children who were quarantined in hospital as a result of SARS often experienced feelings of sadness, attributed to feeling alone, and missing and worrying about family members [10]. There is also some very limited evidence from parental reports in previous epidemics that children suffer even when they are not infected or quarantined. For example, a study of family mental health during the H1N1 epidemic and based on parent reports indicated that 30% of children were experiencing post-traumatic stress [11]. Another study of health care workers during the SARS epidemic reported their perceptions of the negative impact that their work was having on their children, including inducing worries for their parents’ health [9]. However, none of the previous epidemics had such a broad global impact as COVID-19 or resulted in societal changes that had such wide reaching impacts on the lives of children, whether or not they were directly affected by the disease.

### 1.2. Effect of COVID-19 on Children and Adolescents

COVID-19 has resulted in far more widespread impact on the everyday lives of children and adolescents around the world than SARS, MERS or H1N1. For example, the United Nations (UN) Educational, Scientific and Cultural Organisation approximated that school closures affected 862 million children and young people, an estimated half of the global student population [12]. This has led many scholars to express concerns over the likely psychological impact of the COVID-19 pandemic on children and adolescents, e.g., [13,14].

In the absence of evidence on the psychosocial impact of such widespread disruption of children’s lives, research within developmental psychology can highlight where researchers might focus their attention. In addition, there is ample evidence from developmental psychology that the likely impacts will vary significantly depending on the age of the child and the social and demographic characteristics of the family. It also follows that government responses to COVID-19 will affect children of different ages in different ways. School closure impacts significantly on children and adolescents aged between 5 and 18, whereas other measures affect younger age groups. Thus, infants and toddlers may be more affected by the stress that COVID-19 is placing on their parents and there is evidence that over extended periods of time, parental stress may be associated with child behaviour problems in typically developing children [15] and children with developmental disabilities [16]. Although none of the research findings considered in this paragraph relate to the types of short-term disruption caused by COVID-19, they indicate aspects of children’s mental health and wellbeing that might serve as the focus for such research.

The potential for a lack of regular contact with friends to result in loneliness is more likely to be a feature of middle childhood and adolescence [13,14], and is not necessarily mitigated by the use of phones or other forms of communication [17]. Confirmation that these concerns are well founded for young people with a history of mental health problems comes from the findings of UK surveys of young people (13–25 years) while schools and colleges were closed due to COVID-19 and additional lockdown restrictions were in place. The first of these surveys highlights the day-to-day challenges created by school/college closure including the loss of structure and support, the loss of routine and the loss of social connection. A second survey conducted three months later highlighted young people’s perceptions of the challenges for their mental health, including themes of anxiety, loneliness and isolation, and loss of motivation and purpose [18].

These research findings highlight the fact that when schools are closed, adolescents report that there are many aspects of their lives that are disrupted. The impact of long term disruption of this type on mental and physical health is confirmed by research, which indicates that when children are out of school, they are less physically active, spend more time on screens, have more irregular sleep patterns, and less favourable diets, tending to result in weight gain and loss of cardiorespiratory fitness [19]. These negative effects are likely to be exacerbated when lockdown measures result in children being confined to their homes with limited outdoor activities and no interactions with same aged friends [13]. The potential negative impacts for children and adolescents with physical or mental health difficulties are likely to be far more serious, with potential disruptions to their ongoing treatment and support services [20]. This is a particular concern, as there is evidence from longitudinal studies that social isolation in childhood and adolescence carries significant risk of poor adult health, as measured by risk of cardiovascular disease at age 26 years [21] and depression [22]. A rapid systematic review assessed the impact of social isolation and loneliness on the mental health of children and adolescents in the context of COVID-19 and concluded that there would be higher levels of depression and anxiety, both during and after imposed isolation periods come to an end [23].

### 1.3. The Present Study

With the COVID-19 outbreak came many disruptions and alterations to daily life. For children and adolescents, school closures were implemented, affecting an estimated half of the global student population [12]. With social distancing measures and movement restrictions come serious risks of loneliness. This painful emotion has been associated, both in the short and long term, with increased risk of physical ill-health, in addition to higher levels of anxiety and depression in children and adolescents [21,23].

There is a large quantity of research investigating the psychological impact of previous disease outbreaks, including SARS and MERS, on health professionals, hospital employees and adult survivors [24,25,26,27]. In comparison, however, there appears to be a dearth of research assessing the impact of these outbreaks on the mental health of children and adolescents, possibly because they did not involve such widespread closure of schools, child care facilities and other services for children and families Because COVID-19 has resulted in widespread global disruption to such services, it is now imperative to highlight and synthesise the emerging findings of the mental health consequences of this disruption. Findings can be used to inform researchers and youth services about the psychological health of children and adolescents living through the COVID-19 pandemic, and how they can be better supported both during and after the outbreak.

The present systematic review aims to assess the impact of the COVID-19 pandemic on the mental health of children and adolescents. Specifically, the objectives of this review are:

1. To identify mental health outcomes in children and adolescents during the COVID-19 pandemic.
2. To identify correlates, either positive or negative, associated with mental health outcomes in children and adolescents during the COVID-19 pandemic.

## 2. Methods

### 2.1. Design

The present systematic review was conducted in accordance with the Preferred Reporting Items for Systematic Reviews and Meta-Analysis (PRISMA) guidelines [28]. An integrated mixed methods approach was applied including studies with qualitative, quantitative, and mixed methods designs to capture a greater latitude of the field. The protocol of this systematic review was registered with PROSPERO (protocol ID: CRD4202019375). Table 1 lists the inclusion and exclusion criteria.

### 2.2. Search Strategy

Literature searches were conducted in eight databases: PsycINFO, MEDLINE, CINAHL, Scopus, PubMed, EMBASE, ERIC, and the WHO Global Health research database on COVID-19. The latter is a dedicated database summarising all COVID-19 related global research. Searches were refined in accordance with the inclusion criteria using filters to limit results to articles written in English, to peer-reviewed empirical research, to studies conducted in human participants aged 18 years old or younger, and being COVID-19 specific. The searches were performed on June 21st 2020 and no time limit was set. The search string is provided in Supplementary Materials (File S1 and File S2). Because this is an emerging field of research, electronic searches were performed in all fields of the article.

### 2.3. Screening and Data Extraction

**Figure 1:**
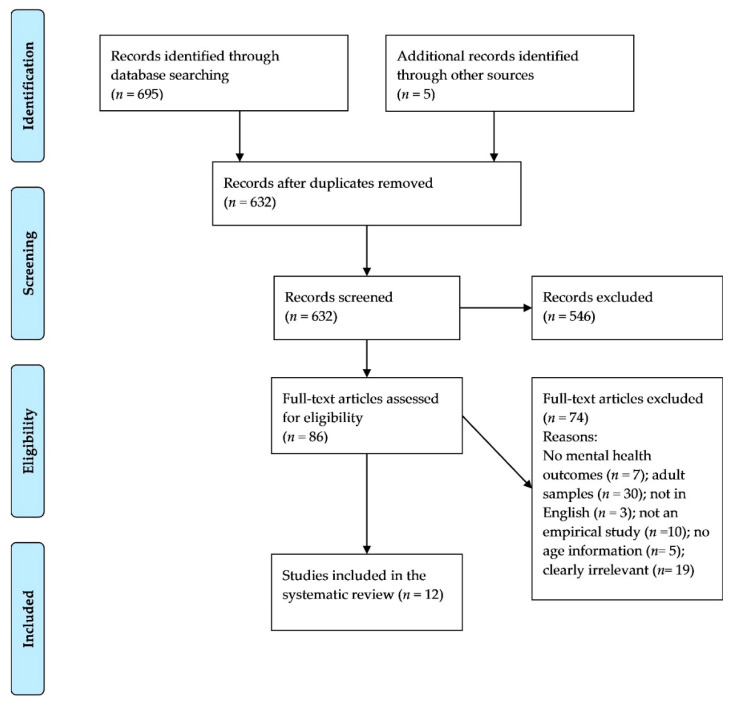
PRISMA 2009 flow diagram.

References that emerged from the database searches were imported to Covidence software (Veritas Health Innovation, Melbourne, Australia) [29]. A total of 700 studies were imported for title and abstract screening and after removing duplicates, 632 studies were retained. Two researchers (F.N., R.N.) performed 100% double title and abstract screening independently with inter-reviewer agreement of 88.1% (*k* = 0.5). Studies that did not meet the inclusion criteria were excluded from full text review and disagreement between researchers was resolved through discussion. A total of 74 studies were deemed eligible for full text review. Studies were subjected to full text review by two researchers independently (F.N., R.N.). The authors of 19 further studies were contacted because the age range of participants was not specified. If authors did not respond within four weeks, the study was deemed ineligible for inclusion. Of the authors that responded, three provided data on the subgroup of young people (<18 years old) and one clarified that their study focused on adults aged 18 years or older. Disagreements after full text review were resolved through discussion, and eight studies were included in the final stage of data extraction. The inter-reviewer agreement was 83.1% (*k* = 0.47). Figure 1 presents the PRISMA flow chart depicting the selection process. As can been seen in Figure 1, studies were excluded for different reasons, while studies that could be assigned a reason due to not meeting inclusion or meeting exclusion criteria were classified as clearly irrelevant. For example, one study that focused on SARS was classified as such.

### 2.4. Quality Assessment

Quality assessment was conducted (*n* = 12) by using the appropriate respective appraisal tool for each research design: Joanna Briggs Institute (JBI) Critical Appraisal Tools [30] and the Mixed Methods Assessment Tool [31] screening criteria. Because all studies were quantitative with a cross-sectional design the JBI Critical Appraisal Tool for Analytical Cross-Sectional Studies and the MMAT Methodological Quality Criteria for Descriptive studies were used. Studies were appraised against the screening criteria of the JBI and MMAT tools by two researchers (FN, RN) independently. Instead of generating an overall score for each study, a qualitative approach was applied by providing a detailed review of the study quality [31]. Studies were appraised as having low, moderate or high methodological quality.

### 2.5. Data Synthesis

Because studies had great heterogeneity in measuring and reporting mental health outcomes, a narrative rather a meta-analytical approach of synthesising findings was deemed appropriate [32].

## 3. Results

### 3.1. Methodological Characteristics of the Studies

Table 2 summarises the characteristics of the included studies. Of the 12 studies that were included in this systematic review, seven were conducted in China, two in Italy, one in Poland, one in Turkey and one in the United States. All studies applied quantitative cross-sectional designs. As expected, due to COVID-19 restrictions, in most studies (*n* = 10) data were collected online, inviting participants to fill in the questionnaires through dedicated survey platforms (*n* = 3), social media platforms (*n* = 4), emails or SMS (*n* = 3), open access forums (*n* = 1) and relevant groups or networks (*n* = 1), while in two studies the data collection procedure was not clearly reported. Data were collected between 28 January 2020 and 20 April 2020 (*n* = 11), while one study did not report exact dates of data collection but only duration [33]. Two studies applied convenience sampling [33,34], one study applied cluster sampling [35], one study recruited students aged 12–18 years old across the country (China) [36], one study applied snowball sampling [37], one study recruited students from four key universities in Wuhan [38], one study recruited students from two primary schools in Hubei province [39], one study recruited participants from another project [40] and four studies provided no or inadequate information on their sampling strategy [41,42,43,44].

Of the 12 studies, seven studies focused explicitly on children and adolescents (age range 3–18 years), four studies were conducted in the general population and one in university students, all five including sub-groups of young people aged 18 years old or younger. Two of the studies that focused on individuals younger than 18 years old used parents as informants. Ten studies included non-clinical samples, while one targeted parents of children diagnosed with autism spectrum disorders (ASD) and one included young patients of an oncology unit who were either receiving treatment or were in the follow up group at the time of the outbreak. In total 12,262 children and adolescents were surveyed, while the sample size varied significantly across studies from 17 to 8072 participants. Information on participants’ gender is inconclusive because five of the 12 studies did not provide any data on gender breakdown. Of those studies that did, four studies reported having more female than male participants in their sample.

In terms of the measures employed by the studies, eight studies used psychometric tools that have been previously standardised and/or established for their psychometric properties, three studies used questionnaires developed by the authors, while one study does not provide information on how the questionnaire was developed [41] (see Table 2 for more details). Of the eight studies that employed standardised measures only three reported reliability coefficients (Cronbach’s alpha) for their samples, which was satisfactory ranging from α = 0.71 to α = 0.95. Quality appraisal using a combination of criteria from two different appraisal tools revealed that most studies were evaluated as of low (*n* = 3) and moderate (*n* = 9) quality.

### 3.2. Mental Health Outcomes

As can been seen in Table 2, mental health outcomes examined by the studies included depression (*n* = 6), anxiety (*n* = 7), psychological distress measured via the Symptom Checklist-90 (*n* = 2), COVID-19 related stress (*n* = 1), fear of contracting COVID-19 (*n* = 2), fear not related to COVID-19 (*n* = 2), worry or concern about contracting COVID-19 (self or family) (*n* = 5), anger and fear about changing daily habits (*n* = 1), somatic symptoms (*n* = 2), obsessive compulsive disorder (OCD) symptoms (*n* = 1), behavioural problems (*n* = 1), burdensomeness (*n* = 1) and emotional reactivity (*n* = 1). One study [43] examined correlates of psychological well-being, such as hope, meaning in life and life satisfaction, one study investigated the relationship of belongingness with mental health outcomes [42], and one study surveyed relationships with peers [40].

#### 3.2.1. Depression

Of the six studies that measured depression in young people, two studies reported mean scores of depression symptoms using Likert point ratings, with higher scores indicating higher levels of depression; three reported prevalence rates, while one did not report any descriptive statistics (see also Table 2 for details). The prevalence of depression in young people across the three studies that reported such information ranged from 22.6% to 43.7% [36,37,39]. One study did not report mean scores of depression in their sample [33].

#### 3.2.2. Anxiety

Anxiety in young people was measured across seven of the included studies. The presence of anxiety symptoms was identified in 18.9% and 37.4% of young people measured by SCARED and GAD-7, respectively, in two studies [36,39]. Four studies reported mean scores of anxiety using Likert-point scales, with higher scores indicating higher levels of anxiety [35,38,42,43], while one study did not report mean scores on anxiety at all [33].

#### 3.2.3. COVID-19 Emotional Reactions and Mental Health Outcomes

Seven studies assessed emotional reactions specific to COVID-19. Of those, two studies did not provide mean scores or other related information [33,37]. Three studies indicated that COVID-19-related emotional reactions are present in children and adolescents by reporting rates ranging from 22% to 62.2%. Specifically, [41] found that approximately 22% of their participants reported fear for the health of relatives; [35] found that almost 40% of primary school children reported being concerned about health and life threats posed by COVID-19; [39] found that 62.2% reported being moderately/quite worried about being infected with the virus. One of the studies [40] conducted in young cancer patients found that almost all of their participants reported feeling a little to moderately afraid of contracting COVID-19 (25 out of 26 young people) as well as a little to moderately afraid of experiencing severe complications (22 out of 26 young people).

Overall, the findings of the included studies suggested that COVID-19 emotional reactions and new social regulations (e.g., social distancing) were associated with a number of negative mental health outcomes in young people. For example, one study [35] found that primary school students reported COVID-19 as a life- and health-threatening disease, which positively predicted somatic and anxiety (but not depression) symptoms in young people during the pandemic. Interestingly, another study showed that fear of COVID-19 significantly predicted depressive, anxiety, and OCD symptoms in adolescents [33]. This study applied sophisticated analyses, in order to gain insight on more complex mechanisms underpinning the impact of COVID-19 fear on mental health outcomes. The findings indicate that COVID-19 related fear associated with negative emotional reactivity may predict depression, anxiety and OCD symptoms in adolescents. Furthermore, it was indicated that anxiety related to expecting challenging circumstances due to Covid-19 pandemic may trigger OCD symptoms [33]. Another study [39] found that worry about being affected by COVID-19 was positively associated with an increased risk of reporting depressive symptoms in children, while being optimistic placed children at decreased risk of depressive symptoms. One study found that adolescents who engaged in social distancing to protect themselves from getting sick and to avoid social judgement reported greater anxiety symptoms. However, those who engaged in social distancing due to peer pressure reported greater depressive symptoms [42]. Finally, a study conducted in children with ASD showed that COVID-19 emergency conditions increased the intensity and frequency of behaviour problems [34].

#### 3.2.4. Psychological Distress and Somatic Symptoms

The two studies that measured psychological distress using the same psychometric tool (i.e., Symptom Checklist-90: SCL-90) found that the majority of their participants aged <18 years old had scores classified within the normal range [37,44]. Somatic symptoms were also assessed by two studies [35,41] with their incidence ranging from 2.39% to approximately 22%. In particular, ref. [35] indicated that primary school students reported mild somatic symptoms measured using the Somatic Self-Report Scale (2.39%), while [41] reported incidence of somatic symptoms of children measured via a study-specific questionnaire using the DSM-5 criteria.

### 3.3. Positive Domains

Positive domains in young people’s lives were explored across three out of the 12 studies included in the present review. These were belongingness [42], relationships with peers [40], hope, meaning in life and satisfaction with life [43]. However, one of those three studies [43] did not conduct separate analysis for the subgroup of young people (<18 years) included in their sample. Feelings of greater belongingness were associated with engaging in social distancing as a result of parental enforcement of rules [42]. Young cancer patients managed to maintain their relationships with peers despite social distancing and self-isolation practices [40].

### 3.4. Age Differences

Of the 12 studies included in this review, two did not report age and gender differences in mental health outcomes. Reported mental health outcomes manifested differently across different age groups of young people and across study samples. One study [41] found that younger children (3–6 years old) were more likely to present with clinginess and fear that family members could contract COVID-19, while older children (6–18 years old) were more likely to show inattention (although marginally) and persistent inquiry. Two studies suggested that older cohorts of young people are likely to report higher levels of symptoms of mental distress. Specifically, [35] found that college students and primary school students differed in levels of anxiety, depression and somatisation, with primary school students reporting milder mental health symptoms. Concerns about the threat that COVID-19 poses to life and health was the only significant predictor of somatic symptoms in the younger cohort. Similarly, in an adolescent sample (12–18 years), older adolescents (senior high school) were more likely to report higher depression and anxiety symptoms than their younger counterparts (junior high school) [36]. One study showed that as age increases, the intensity of behaviour problems induced by the pandemic in children with ASD decreases [34]. One study found that those younger than 18 years old (*n* = 22) had significantly increased likelihood of appearing in the high-risk group, in terms of reported psychological distress, when compared to other age groups of the study sample [44].

Four studies found no evidence associating age with mental health symptoms. Specifically, one study conducted in the general population but including a subgroup of individuals younger than 18 years (*n* = 34) found that this younger group did not differ from older groups in the reported levels of psychological distress [37]. Depression scores indicated differences across age groups, but the analyses of this study did not specify between which groups these differences lie. Similarly, another study conducted in the general population suggested that the youngest cohort of participants (<18 years old, *n* = 17) did not differ in any of the outcome measures, including anxiety [43]. No age differences were reported in the two studies focused exclusively on adolescent samples [39,42].

### 3.5. Gender Differences

Of the four studies that examined gender differences in young people under 18 years of age, differences were evident in two studies [36,42], but only one study reported explicitly that females were more likely to report higher levels of depression and anxiety [36]. The two remaining studies found no evidence of gender differences [35,39].

## 4. Discussion

This systematic review aimed to add to our knowledge by evaluating and synthesising existing evidence on the impact of COVID-19 on the mental health of young people aged 18 years old or younger. We performed searches in eight databases, and after completing the screening process, we included 12 studies for data extraction. Because studies were greatly heterogenous in measuring and in reporting mental health outcomes, a narrative rather a meta-analytical approach of synthesising findings was deemed appropriate. Quality appraisal indicated that studies were evaluated as being of low or moderate quality.

Six of the included studies examined mental health outcomes by applying basic descriptive analyses and six conducted further analyses to examine the significance of variables such as demographic characteristics and COVID-19-related emotional reactions in predicting mental health outcomes (see Table 1 for more details). Two of the included studies aimed to deepen our understanding of the nature of mental health symptoms by applying more sophisticated analyses [33,43]. However, one of those studies [43] focused on adults while also including young people in their sample, yet did not perform analyses by age group. Hence, only one of these conducted analysis in youth [33]. This study showed that COVID-19 fear may trigger OCD symptoms in young people, while emotional reactivity, depression, anxiety, and experiential avoidance may help better explain the relationship between COVID-19-related fear and OCD symptoms in young people. Five studies targeted the general population while including subgroups of young people under 18 years old. Of those five studies (see also Table 2), four included very small samples (*n* = 17–34), which warrants caution in interpreting their findings because of the limited statistical power. One study focused on university students included *n* = 1118 young people < 18 years old; however, no findings other than descriptive statistics are reported [38].

### 4.1. Impact of COVID-19 on Youth Mental Health

The overall findings of this review indicate that the COVID-19 global pandemic has impacted young people’s mental health. While COVID-19 emotional reactions were associated with a number of mental health outcomes, the reported rates across studies did not allow for inferences regarding levels of these mental health outcomes associated with the pandemic per se. Specifically, three studies reported rates of depression symptoms ranging from 22.6% to 43.7%, while two studies reported rates of anxiety symptoms ranging from 18.9% to 37.4% in young people during the pandemic. Other pre-COVID 19 studies reported similar or even higher depression levels in young people [45]. However, it is argued that anxiety rates may be higher than the rates reported in other pre-COVID-19 studies [39,46]. Nevertheless, interpretation of these findings warrants caution as further empirical research is required in this field.

Two studies included in this review measured young people’s reported levels of psychological distress [37,44], and one of these studies proposed that being under 18 years of age carries an increased likelihood of being in the high-risk group [44]. However, both studies focused mainly on adults (>18 years old), including very small samples of youths < 18 years old (*n* = 34 and *n* = 22), thus any inference regarding levels of psychological distress could be redundant. Two studies reported rates of somatic symptoms in children ranging from 2.39% to 22% [35,41]. Somatic symptoms are common in children and adolescents with prevalence rates ranging approximately from 10% to 30% [47,48]. This discrepancy in reported rates may be because one of the included studies used a self-report established measure [35], while the other study [41] used a study-specific measure with parents as informants. Hence, these rates should be interpreted individually within the study context and not as aggregated findings.

Despite the lack of sufficient evidence on reported prevalence rates, the findings of this review do offer an insight on the associations between COVID-19-related emotional reactions and mental health outcomes in young people. Most of the included studies showed that COVID-19-related emotional reactions such as worry, fear about contracting the virus and stress predicted mental health outcomes in young people such as depression, anxiety, OCD symptoms, somatic symptoms and intensified behaviour problems. This is generally consistent with evidence on the impact of COVID-19 on adult mental health [49,50], yet not surprising. It has been well established in the literature that children, and especially adolescents, are susceptible to experiencing mental health problems, while most mental disorders have their onset in this age period [51,52]. For example, somatic symptoms in youth may be related to their new social reality due to COVID-19, which possibly introduced new challenges to young people in addition to the developmental challenges of their age. Furthermore, this new situation with the imposed social and physical restrictions may have also introduced additional barriers to accessing informal and formal help-seeking for mental health problems in youths, similarly to adults [53,54]. In addition, young people may now have restricted exposure to elements that operate as protective agents against mental health difficulties. For example, research shows that an adult figure other than a parent (such as a teacher, coach, etc.) or engagement with community activities can serve as informal sources of mental health help-seeking and as positive elements, respectively, in young people’s lives, which may help them cope with mental health difficulties [55,56]. Hence, children and adolescents may experience increased levels of mental health problems in the light of a global pandemic.

One of the included studies explored adolescents’ motivation to engage in social distancing in relation to reported depression and anxiety levels during the COVID-19 pandemic [42]. This study suggested that adolescents who engaged in social distancing to protect themselves from getting sick and to avoid social judgement reported greater anxiety symptoms. However, those who engaged in social distancing due to peer pressure reported greater depressive symptoms. Notably, motivation to engage in social distancing due to complying to parental or governmental rules was not associated with any mental health outcome. Peer pressure has been linked to depression and anxiety in adolescents [57], thus when social distancing is perceived as a result of peer pressure, it may lead to experiencing depression and anxiety. In contrast, perceiving social distancing as a family rule may enhance the sense of family cohesion, which has been identified as a contributing factor to positive mental health outcomes in adolescents [58]. Furthermore, because family support has been identified as a protective factor associated with resilience in youths exposed to adversities e.g., [55,59], in the presence of an adversity such as living amidst a pandemic, family cohesion may also protect against distress.

Despite emerging evidence showing that clinical manifestation and prognosis of COVID-19 infection may be milder in paediatric cancer patients than in adults, immunosuppressed paediatric patients are treated as a high risk group [60,61]. Recommendations of international organisations indicate that children, adolescents and young adults who are cancer survivors may be at increased risk of experiencing severe complications of COVID-19 infection [62]. These may cause additional distress in young cancer patients. Indeed, one of the included studies surveyed mental health outcomes in young cancer patients who were either receiving treatment at the time or were patients in follow up who had completed their treatments [40]. Of those young people who were under 18 years old, almost all reported feeling a little to moderately afraid of contracting COVID-19 (25 out of 26), while 85.6% reported feeling a little to moderately afraid of experiencing severe complications. In addition, 61.5% reported feeling moderately worried about their family becoming ill.

Available research shows that COVID-19 restrictions introduced additional challenges to children with intellectual and developmental disabilities (IDD), including ASD, and their parents, e.g., [63]. One of the studies included in the present review [34] investigated the impact of the COVID-19 crisis on children with ASD and showed that young people with pre-existing behaviour problems are twice as likely to exhibit behaviour problems of increased intensity and frequency during the pandemic. Maintaining a daily routine with structured activities and being supported by the provision of specialised educational and healthcare services is of essence for children with ASD and their parents alike [64]. COVID-19 emergency restrictions, including school, day-care and after-school closures as well as mandatory long-term lockdowns, disrupted this structured routine for families with children with IDD/ASD, thus making the management of everyday life extremely challenging. This was reflected in parents’ reported needs for the provision of health care and especially in-home services [34].

### 4.2. Age and Gender Differences

There was some evidence suggesting that the impact of COVID-19 may differentiate across age groups of young people regarding the type and levels of the reported mental health outcomes. Two studies indicated that as age increases, reported levels of anxiety and depression are likely to increase as well [35,36]. These findings highlight the importance of considering developmental differences in responses to COVID-19. This is also consistent with evidence from other pre-COVID studies indicating that middle and late adolescents are likely to experience more heightened levels of distress than their younger peers [65,66]. International trends suggest that older adolescents tend to use social media as well as electronic media communication more frequently than younger adolescents [67], which may have resulted in being exposed to any kind of COVID-19-related information more frequently and intensely. Frequent social media exposure during this pandemic has been associated with increased likelihood of depression and anxiety in adults [68]. Because older adolescents use social media more frequently than their younger peers and exposure to social media has been linked with increased likelihood of experiencing depression and anxiety in adults during this pandemic, older adolescents may experience higher levels of distress than younger adolescents.

One study indicated that younger children (3–6 years) experience different mental health symptoms than their older counterparts (6–18 years) [41]. Despite its merit, this finding should be interpreted with caution because of treating young people of discrete developmental stages as one cohort (6–18 years) in addition to a lack of reported methodological clarity. Two other studies that focused on adolescent samples reported no age differences [39,42]. These contradictory findings regarding age differences may be because studies that examined age differences within an adolescent sample may not have had a wide enough range to identify developmental differences. No conclusions can be drawn from the studies with very small sample sizes of young people [37,40,43,44]. Collectively, making any further inferences on the age and gender differences based on the findings from the included studies was deemed inappropriate due to their great methodological heterogeneity.

### 4.3. Strengths and Limitations

This research offered a systematic review and synthesis of the evidence on COVID-19’s impact on youth mental health, focusing on children and adolescents (18 years old or younger). This review highlighted considerable implications in relation to the impact of the COVID-19 pandemic on youth mental health. Young people both with and without an underlying mental and/or physical health condition seem to be affected by the pandemic and experience feelings and emotions similar to those experienced by adults. This needs to be considered when updating the provision of youth mental health services in light of the pandemic, targeting children and adolescents up to 18 years old as well as their parents. For example, one of the studies included showed that parents of children with ASD need more specialised support to tackle the disruption of their families’ structured daily routines [34].

This systematic review has a number of limitations. First, the included studies were found to have low to moderate methodological quality regarding their sampling method, unclear validity of measurement and statistical analysis. Hence, this needs to be considered when interpreting the findings. Second, we excluded a number of studies from our analyses, due to lacking and/or unclear information. Third, we included only studies published in English. Thus, some studies meeting the inclusion criteria may have not been included in the review. Fourth, we were only able to include a limited number of studies due to the fact that a limited number of studies have been published because COVID-19 was only declared a pandemic in March 2020. These limitations should be addressed in future research.

### 4.4. Recommendations

COVID-19-related youth mental health research is an emerging field of evidence and our systematic review highlighted important methodological gaps providing some directions for enhancing rigour in future research. First, there is a need for more studies focusing on children and adolescent samples rather than targeting the general population and including subgroups of young people. These studies focusing on the general population include either small sub-samples of young people and/or use psychometric tools that may not be appropriate for youth populations. For example, there is lacking evidence on whether the Somatic Self-Rating Scale is appropriate for use in young children, as it was developed for use in adult clinical populations [35]. Similarly, some studies used questionnaires developed for the study purposes without any evidence that these are psychometrically sound for use in specific age cohorts [38,40]. In addition, studies should use validated and standardised psychometric tools that are appropriate for assessing mental health outcomes in youth rather than using single items or study-specific questionnaires developed by the authors without providing evidence of their psychometric quality. Second, age and gender differences were not examined in depth across most of the studies. Because there is evidence on the importance of incorporating these demographic variables in informing policy decisions and the provision of services [69,70], future studies should also incorporate those in their research designs. Finally, we observed a lack of clarity and detail of information that is normally required in empirical studies. This is maybe due to the time constraint and the urgent need to provide the first empirical evidence regarding a continuously developing and emerging field of research. Addressing these will contribute to generating more robust evidence on the impact of COVID-19 on youth mental health that will add to our knowledge as well as help inform policy decisions regarding the provision of mental health services and the delivery of more targeted mental health prevention/intervention programmes.

## 5. Conclusions

International health organisations have warned governments to be prepared to tackle the mental health complications associated with COVID-19. While there is increasing empirical evidence indicating the mental health complications of COVID-19 in adults, our knowledge of the impact of the pandemic on youth mental health remains significantly restricted. This may be because children and adolescents, especially those younger than 18 years old, are likely to present with milder clinical features (or even as asymptomatic) and prognosis of the infection than adults. Hence, most research to date has focused on exploring the mental health consequences in older rather than in younger cohorts. We extracted data from 12 studies that examined mental health outcomes in children and adolescents < 18 years old. The findings indicate that COVID-19 has an impact on youth mental health and is particularly associated with depression and anxiety in adolescent cohorts. This is not surprising considering that psychological distress associated with depression and anxiety is highly prevalent in adolescents aged between 12 and 18 years old [71,72]. However, the COVID-19 pandemic was associated with other mental health difficulties as well, such as OCD and somatic symptoms, psychological distress and increasing behavioural difficulties. Specifically, emotional reactions to COVID-19, such as stress, fear, worry, and concern predicted mental health outcomes in young people. Interestingly, it has been suggested that some pre-COVID-19 studies reported similar or even higher depression levels in young people, which may indicate that the presence of mental health problems in young people may not be only due to the pandemic. Studies included in this review indicated a lack of methodological quality, clarity, and rigour. Subsequently, it is imperative to generate more empirical evidence using validated psychometric tools and more robust research designs, including designs from the qualitative paradigm. Finally, to capture the developmental aspects of the impact of COVID-19 on youth mental health, future research should be inclusive of diverse developmental cohorts of young people such as children, early, middle, and late adolescents.

## Figures and Tables

**Table 1:** Inclusion and exclusion criteria.

Inclusion Criteria	Exclusion Criteria
Quantitative, qualitative, mixed methods studies Human studies Peer-reviewed papers Mental disorders Neurodevelopmental disorders/any other disorder Any mental health outcome COVID-19 related research	Not in English Studies [that only included] of adults 18 years old or older Studies that did not report age

**Table 2:** Methodological features and quality appraisal of included studies.

Authors	Country	Date of Data Collection	Sample Characteristics	Sampling Strategy/Data Collection Method	Measures	Analytical Approach	Mental Health Outcomes and Correlates	Quality Appraisal
Casanova et al., 2020 [40]	Italy	2–7 March 2020	Pediatric oncology patients adolescents and young adults, *n* = 26 < 18 years (14 males) receiving treatment *n* = 10 follow up after completed treatment	Directly contacted participants recruited for other projects, data collected by email	Questionnaire including items developed by authors, no reliability other information reported	Descriptives	Fear of contracting COVID-19 and of implications; worry about family; relationships with peers; anger and fear about changing daily habits	Low
Colizzi et al., 2020 [34]	Italy	6–20 April 2020	*n* = 527 children diagnosed with autism spectrum disorders mean age = 13 year, *SD* = 8.1, no age range reported, no information on gender	Convenience sampling online parent survey distributed by healthcare professionals, autism advocacy and family support networks	Self-reported diagnosis of ASD; 40-item questionnaire developed for study purposes using focus groups (no reliability reported for the present sample)	Descriptives; Logistic Regressions	Well-being measured via frequency and intensity of behavioural problems	Medium
Jiao et al., 2020 [41]	China	Second week of February 2020	*n* = 320 children and adolescents aged 3–18 years old (168 females), no other information reported	Parent survey, no other information	Questionnaire incorporated DSM- 5 criteria; no reliability or other information reported	Descriptives; Age group differences examined no information reported on statistical tests used	Poor sleep; nightmares; poor appetite; physical discomfort and agitation; inattention; clinginess; worry; irritability; fear of the health of relatives; obsessive request for updates; sleeping disorders	Low
Liu, Liu et al., 2020 [36]	China	February and March, 2020	*n* = 209 primary school students (5th and 6th grade; 116 females) no other age information	Cluster sampling (unclear whether how data collection was conducted)	SSS; (no reliability reported for the present sample)	Descriptives, *T*-tests, Kruskal–Wallis test; Spearman rank correlations; logistic regressions	Concerns regarding COVID-19; somatic symptoms; depression; anxiety	Medium
Liu, Luo et al., 2020 [37]	China	30 January to 3 February 2020	*n* = 608 adults, *n* = 34 < 18 years old, no information on age range and gender	Snowball sampling, online via social media platforms	SDS; SCL-90 (reliability not reported for the present sample) single item measuring COVID-19 worry	Descriptives; *T*-tests; ANOVAs; chi-square	Worry about contracting COVID-19 depression; ‘Psychological abnormalities’	Medium
Oosterhoff et al., 2020 [42]	United States	28–29 March 2020	*n* = 683 adolescents, mean age = 16.35, *SD* = 1.13, range 13–18 years, 75.3% females	No information on sampling strategy, online via social media platforms,	PROMIS anxiety scale; PROMIS depression scale; INQ burdensomeness; INQ Belongingness (reliability not reported for all measures for the present sample)	Descriptives; correlations regressions	Anxiety; depression; burdensomeness; belongingness	Medium
Seçer et al., 2020 [33]	Turkey	Data collected during 15 days, no other information reported	*n* = 568 adolescents, mean age = 16.4, *SD* = 2.14, age range 14–18 years (61.1% males)	Convenience sampling online via the provincial education directorate using social media apps and emails	OCI- Child Version; ERS; DAS-CV; Fear of COVID-19 Scale;	Confirmatory factor analyses; structural equation modeling	Obsessive-compulsive symptoms; emotional reactivity; depression; anxiety; fear of COVID-19	Medium
Tian et al., 2020 [44]	China	31 January to 2 February 2020	*n* = 1060 adults, *n* = 22 < 18 years old, no information on gender	Online via the Wenjuanxing survey platform, no other information provided	SCL-90 using the Global Severity Index (reliability not reported for the present sample)	Descriptives; *T*-tests; Anovas	Psychological distress through the nine dimensions of SCL-90	Medium
Trzebiński et al., 2020 [43]	Poland	1–4 April 2020	*n* = 317 adults, *n* = 17 < 18 years, no information on gender	Online via open access forums, no other information provided	COVID-19 SS; STAI; SWLS; MIL; BH	Correlations, Between groups comparisons Correlations and Mediation analysis (total sample)	COVID-19 stress; state-trait anxiety; satisfaction with life; meaning in life; hope	Medium
Xie et al., 2020 [39]	China	28 February to 5 March 2020	*n* = 1784 primary school students grades 2 through 6 (56.7% boys)	Online via the Wenjuanxing survey platform, two primary schools in Hubei province	CDI-S; SCARED (reliability not reported for the present sample)	Descriptives Generalised and Logistic regressions	Worry about being infected with COVID-19; Anxiety; Depression	Medium
Yang et al., 2020 [38]	China	28 –30 January 2020	*n* = 8252 university students, *n* = 1118, mean age = 17.9, SD = 0.30, range 16–18 years, no information on gender	Online survey via social media, sms, email, four key universities in Wuhan	A five-point Likert scale created by authors to measure levels of anxiety and fear	Descriptives	Anxiety; Fear	Low
Zhou et al., 2020 [36]	China	8–15 March 2020	*n* = 8140 students invited (median age 16, range 12–18 years), *n* = 8072 included in the study (4326 females)	Online via the Wenjuanxing survey platform, junior and senior high school students in China aged 12–18 years	PHQ-9; GAD-7 (reliability not reported for the present sample)	Desrciptives; Chi-square tests; *T*-tests; Logistic regressions	Depression; Anxiety	Medium

ASD, Autism spectrum disorders; BH, The Basic Hope Scale; CDI-S, Children’s Depression Inventory—Short Form; COVID-19 SS, COVID-19 Stress Scale; DAS, Depression and Anxiety Scale for Children; ERS, Emotional Reactivity Scale; Fear of COVID-19 Scale; GAD-7, Generalized Anxiety Disorder Scale-7 Chinese version; INQ, Interpersonal Needs Questionnaire; MIL, The Meaning in Life Scale; OCI, Obsessive Compulsive Inventory - Child Version; PHQ-9, Patient Health Questionnaire; PROMIS, Patient-Reported Outcomes Measurement Information System scale; SCARED, Screen for Child Anxiety Related Emotional Disorders; SCL-90, Symptom Checklist-90; SDS, Self-rating Depression Scale; SSS, Somatic Self-Rating Scale; STAI, State Trait Anxiety Inventory; SWLS, Satisfaction with Life Scale.

## Supplementary Materials

The following are available online at https://www.mdpi.com/1660-4601/17/22/8479/s1, File S1: PRISMA 2009 Checklist; File S2: Example of original search string for PsycInfo.
