# Supplementary material for: Exploring the Impact of COVID-19 on Mental Health Outcomes in Children and Adolescents: A Systematic Review
Source: Int J Environ Res Public Health. 2020 Nov 16;17(22):8479. doi: 10.3390/ijerph17228479 (PMC7698263; doi:10.3390/ijerph17228479)
Supplement: Supplementary file 1 [file ijerph-17-08479-s001.zip › S2 file.docx]

Supplementary 2. Example of original search strategy for PsycInfo on June 21st 2020

(covid* OR coronavirus* OR "novel coronavirus" OR nCOV OR quarant*) AND (psychiatr* OR psycholog* OR mental OR "mental health" OR "mental illness" OR "mental outcomes" OR “mental disorder” ) AND (child* OR adolescen* OR young OR youth OR teen* OR infant OR puberty) AND (survey* OR quant* OR qual* OR questionnaire) AND (depress* OR anxiety OR stress* OR "posttraumatic stress" OR PTSD OR wellbeing OR well-being OR mood* OR insomnia)

Language limited to English. Publication type limited to peer-reviewed papers. Research in animals was excluded.
